# Chemical carcinogens used to induce spontaneous tumors may suppress tumor specific T-cell immunity

**DOI:** 10.1186/2051-1426-1-S1-P217

**Published:** 2013-11-07

**Authors:** Dan Herendeen, Ekram Gad, Lauren Rastetter, Dominick Auci, Ben Curtis, Marlese Koehnlein, Mary L Disis

**Affiliations:** 1 University of Washington, Seattle, WA, USA

## 

Many mouse models of breast cancer have been developed that closely replicate human breast cancer subtypes at the genomic level. In order to develop vaccines that could prevent a broad spectrum of breast cancer subtypes, a combination of different spontaneous tumor models are needed. Transgenic models are available that mimic HER2+/luminal breast cancer (TgMMTV-Neu) and triple negative/basal-like breast cancer (TgC3(I)-Tag). Heterogeneous tumor types, including ER+ breast cancers, can be induced by treating mice with medroxyprogesterone (MPA) and the carcinogen 7,12-dimethylbenz[a]anthracene (DMBA). The potential disadvantage of using the DMBA model to study vaccine efficacy is that DMBA has been reported to be an immunotoxin, potentially affecting the function of T, B, and antigen presenting cells. In order to address the utility of the DMBA model for vaccine studies, we performed experiments to look at the effect of DMBA on the immunogenicity of breast cancer vaccines. To assess immunity, three groups of FVB mice (n=8 each) were injected with either (a) adjuvant only, (b) IGF-IR peptide vaccine, or (c) IGF-IR peptide vaccine followed by MPA/DMBA treatment. Seven days following the final DMBA treatment (49 days post-vaccination) splenocytes and sera were subject to immunological testing. IFNγ ELISPOT assays and ELISAs were performed to measure IGF-IR peptide-specific T-cell and humoral immunity, respectively. ELISPOT assays revealed that DMBA treatment significantly reduced-T cell responses to the vaccine peptides (22% compared to the vaccine-only group, p=0.015), but had no significant effect on T cell responses to the positive control mitogen ConA (p=0.52) or background activity (p=0.87). For the control, vaccinated and vaccinated/DMBA groups, average spots/well with IGF-IR peptides were 0.8 (±0.9), 38.7 (±27.4), and 8.6 (±6.3), and with ConA were 352 (±30.5), 325( ±39.6), and 242 (±73.2), respectively. Humoral immunity was not impaired in the DMBA treated animals. Two IGF-IR peptides (p1212 and p1302L) generated significant antibody responses in the vaccinated and vaccinated/DMBA animals compared to the control group (p=0.001 to 0.018), but there was no significant difference between the vaccinated ±DMBA groups (p=0.58 and 0.72). Mean IgG titers against p1212 were 0.07 (±0.08), 167 (±0.74), and 144 (±91) μg/ml, and against p1302L were 0.02 (±0.04), 129 (±89), and 146 (±98) μg/ml for the control, vaccinated, and vaccinated/DMBA groups, respectively. Chemically induced spontaneous tumor models are commonly used for studies of lung, skin, and colon cancers. Data presented here would suggest an evaluation of the carcinogens impact on immunity should be undertaken prior to using such models to evaluate immune therapeutics.

